# 

**Published:** 1894-10-06

**Authors:** 


PRICE TWOPENCE.
No. 419, Vol. XVII.—Oct. 6th, 1894.
[Registered at the General Post Office as a Newspaper for transmission in the United Kingdom and Abroad.]

WITH EXTRA NURSING SUPPLEMENT.

Per Annum, 10s. 6d., post free.

Monthly Parts, 6d.; post free, 8d.

Ditto per Annum, 8s. 6d. post free.

### THE HOSPITAL

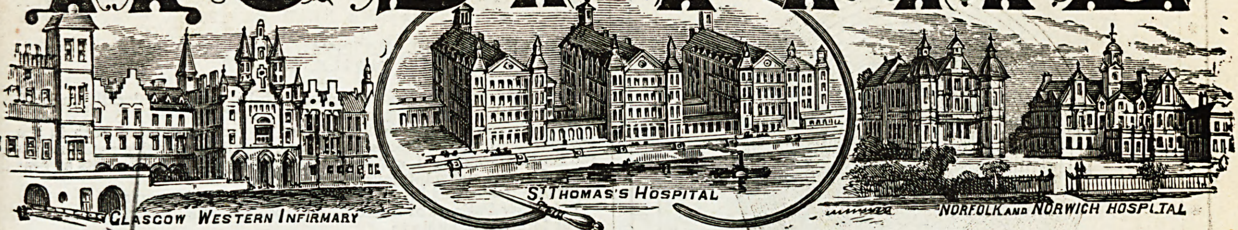

No. 419, Vol. XVII. WITH EXTRA NURSING SUPPLEMENT. SATURDAY, OCT. 6TH, 1894.

